# Orbital Radiotherapy for Thyroid-Associated Orbitopathy in the Era of Targeted Biologics: Reposition, Not Replace

**DOI:** 10.3390/medicina62091671

**Published:** 2026-08-31

**Authors:** Ji Hwan Jo, Ki Ho Seol

**Affiliations:** 1 Department of Radiation Oncology, Daegu Catholic University School of Medicine, Daegu 42472, Republic of Korea; d240063@cu.ac.kr; 2 Department of Radiation Oncology, Daegu Catholic University Medical Center, Daegu 42472, Republic of Korea

**Keywords:** thyroid-associated orbitopathy, Graves orbitopathy, thyroid eye disease, orbital radiotherapy, teprotumumab, glucocorticoids, diplopia, cost-effectiveness

## Abstract

*Background and Objectives*: Orbital radiotherapy (OR) has been used for moderate-to-severe active thyroid-associated orbitopathy (TAO) for decades, yet its role is repeatedly questioned. Sham-controlled randomized trials have not demonstrated a benefit of OR on proptosis or soft-tissue change, whereas clinical practice guidelines continue to recommend OR, combined with glucocorticoids, as a second-line option, particularly for diplopia and restricted ocular motility. The insulin-like growth factor 1 receptor (IGF-1R) inhibitor teprotumumab, with marked effects on proptosis and diplopia, has prompted some to regard OR as obsolete. This review aims to reconcile the long-standing discordance between randomized-trial evidence and guideline recommendations and to reposition OR within the contemporary, biologics-inclusive treatment algorithm. *Materials and Methods*: We performed a semi-systematic narrative review of PubMed and Embase (inception to June 2026), prioritizing randomized controlled trials, comparative and large observational studies, current guideline and consensus documents, and recent systematic reviews and meta-analyses across radiobiology, dose and technique, efficacy, glucocorticoid combination, safety, health economics, and biologics. *Results*: The randomized endpoints that failed (proptosis and soft-tissue swelling) are not those that OR is mechanistically expected to improve, whereas its benefit for motility and diplopia aligns with the indication guidelines specified; meta-analyses support a motility and combination benefit. Against teprotumumab, OR shows a smaller proptosis effect but established long-term safety, wide accessibility, and far lower cost, while teprotumumab’s real-world durability is limited and its cost and adverse-event burden is substantial. *Conclusions*: OR is best understood not as a competitor to be replaced but as an accessible, low-cost, motility-directed modality with real limitations of its own (a modest effect on proptosis and a frequent residual need for surgery), whose optimal sequencing alongside glucocorticoids and biologics has yet to be defined by head-to-head and cost-effectiveness studies.

## 1. Introduction

Thyroid-associated orbitopathy (TAO), also termed Graves’ orbitopathy or thyroid eye disease, is the most common extrathyroidal manifestation of Graves’ disease and follows a characteristic course of an active inflammatory phase followed by an inactive fibrotic phase. Management of the moderate-to-severe active phase has long relied on immunomodulation, with glucocorticoids as first-line therapy and orbital radiotherapy (OR) positioned as an adjunct.

Current guidelines retain a defined place for OR. The 2021 European Group on Graves’ Orbitopathy (EUGOGO) clinical practice guidelines recommend OR, in combination with glucocorticoids, as a second-line treatment for moderate-to-severe active disease, particularly in patients with diplopia and/or restricted extraocular motility (Recommendation 16; recommendation strength 1, moderate-certainty evidence) [1]. By contrast, the 2022 ATA/ETA consensus positions teprotumumab as first-line therapy for most moderate-to-severe active disease, particularly when exophthalmos predominates—a notable divergence from EUGOGO that itself signals that the optimal positioning of the available modalities is unsettled [2].

This established position has been disrupted from two directions. First, the IGF-1R-targeting monoclonal antibody teprotumumab produced a proptosis response in 83% of patients versus 10% with placebo, and a diplopia response in 68% versus 29%, at week 24 in the pivotal OPTIC trial [3], leading to enthusiasm that biologic therapy might supersede older modalities. Second, and in tension with guideline endorsement, several sham-controlled randomized trials failed to show that OR improves proptosis or soft-tissue changes.

Two gaps follow. The discordance between randomized-trial results and guideline recommendations is rarely reconciled in a manner useful to the clinician, and the relative position of OR has not been re-examined now that the durability and cost limitations of teprotumumab are becoming clear from real-world, post-approval experience. The distinct contribution of this review is to address these two gaps together: to reconcile the trial–guideline discordance on mechanistic grounds and to reposition OR specifically against contemporary teprotumumab data, rather than to re-catalog the modality in isolation as earlier reviews have done. We synthesize the mechanism, technique, efficacy, combination use, and safety of OR, engage the strongest arguments against it, and propose how it should be positioned, rather than retired, within a biologics-inclusive algorithm. The repositioning argument advanced here rests on four claims, which the remainder of this review develops in turn: (i) the benefit of OR is motility- and diplopia-directed rather than proptosis-directed, which is why sham-controlled trials built around proptosis endpoints did not detect it; (ii) that benefit is activity-dependent, so patient selection by inflammatory activity matters more than time since diagnosis; (iii) OR retains a decisive advantage in cost and accessibility that is material wherever biologic therapy is constrained; and (iv) OR and biologics are better understood as sequential and complementary than as mutually exclusive.

## 2. Materials and Methods

This narrative review was conducted along semi-systematic lines to limit selection bias. By semi-systematic, we mean that the search strategy, eligibility criteria, and screening process were specified in advance and are reported below, but that no quantitative pooling, no formal risk-of-bias instrument, and no PRISMA flow accounting were applied. The result is a structured narrative synthesis, not a systematic review or meta-analysis.

*Search.* We searched PubMed and Embase from inception to June 2026 for English-language articles, combining terms for the condition (“Graves’ orbitopathy”, “thyroid eye disease”, “thyroid-associated orbitopathy”) with “orbital radiotherapy” or “orbital irradiation” and, in successive searches, with terms for radiobiology and mechanism, dose and fractionation, technique (IMRT, VMAT, proton), glucocorticoids and biologics (teprotumumab, rituximab, tocilizumab), magnetic-resonance predictors, dysthyroid optic neuropathy, adverse effects (retinopathy, cataract, malignancy), quality of life, and cost-effectiveness. Reference lists of identified reviews were hand-searched.

*Eligibility criteria.* Eligibility was defined as follows:

- *Population.* Adults with thyroid-associated orbitopathy. Studies of moderate-to-severe active disease were prioritized; reports confined to mild or inactive disease were used only for background.
- *Intervention.* OR at any total dose, fractionation, or technique, delivered alone or in combination with glucocorticoids or biologic therapy.
- *Comparators.* Sham irradiation, no radiotherapy, glucocorticoids alone, biologic therapy alone, or an alternative radiotherapy schedule. Single-arm reports were eligible where they addressed safety, technique, or health economics, for which comparative data are scarce.
- *Outcomes.* Composite treatment response, clinical activity score, diplopia and ocular motility, proptosis, dysthyroid optic neuropathy, quality of life (GO-QoL), adverse effects (radiation retinopathy, cataract, secondary malignancy), and cost or cost-effectiveness.
- *Study design.* Randomized controlled trials of any size; comparative observational studies; non-comparative observational cohorts of at least 100 patients; current guideline and consensus documents (EUGOGO 2021; ATA/ETA 2022); and systematic reviews or meta-analyses. Case reports and case series of fewer than ten patients were excluded, except where they were the only available evidence for dysthyroid optic neuropathy. Non-English reports and articles without original or directly relevant data were excluded.

*Definition of disease activity in the included studies.* Activity was not defined uniformly across the literature. Most randomized trials and cohorts classified activity by the clinical activity score, with thresholds varying between studies; a smaller number used orbital magnetic resonance imaging, chiefly T2-weighted short-tau inversion recovery signal intensity or T2 relaxation time, either as the sole criterion or as an adjunct to the clinical activity score. We did not impose a single activity definition retrospectively, since doing so would have excluded most of the randomized evidence. Where the distinction affects interpretation, we state the definition used by the source in the text, and the heterogeneity of activity assessment is acknowledged as a limitation in Section 11.

*Hierarchy of evidence and treatment of small studies.* The 100-patient threshold applied only to non-comparative observational cohorts, where a small sample carries little information about effect size. Randomized trials of any size and comparative observational studies of any size were eligible, because in this field, the pivotal randomized trials are themselves small. A small number of additional sources with fewer participants are cited not as efficacy evidence but for a single, narrowly scoped observation that no larger dataset yet provides; in each such case, the sample size is stated in the text so that the reader can weigh the claim accordingly. The 21-patient retrospective analysis discussed in Section 9, for example, is used only for its observation on the durability of the teprotumumab proptosis response, and its size is stated where it is cited.

*Screening.* Titles and abstracts were screened independently by both authors. The authors then reviewed the complete set of retrieved articles together and agreed by consensus both on which studies to include and on how the retained material would be organized across the sections of this review; any disagreement over inclusion was resolved during that discussion. Both authors subsequently obtained and examined the full text of every included reference, verifying each bibliographic record against PubMed and confirming that the figures, effect estimates, and outcome definitions cited here matched the source publication.

*Handling of overlapping syntheses.* Several systematic reviews and meta-analyses in this field draw on the same small set of randomized trials. To avoid presenting the same primary data as if it were independent corroboration, we cite the most recent and most comprehensive synthesis for a given question and, where a second synthesis rests on substantially the same primary trials, we say so in the text rather than listing both as separate support. Where a pooled estimate depends on only two or three contributing trials, that number is stated.

*Qualitative appraisal.* No formal quality-appraisal instrument was applied. Instead, for each randomized trial, we noted sample size, blinding, single- versus multi-center design, and whether the primary endpoint was met; for meta-analyses, we noted the number of contributing trials for each pooled estimate; and for observational studies, we noted whether outcomes were ascertained clinically or by administrative coding. These features are reported in the text where they affect the weight a claim can bear.

Because outcome measures and reporting were heterogeneous, no quantitative pooling was performed, and the breadth of topics, from radiobiology to health economics, is covered selectively rather than exhaustively.

## 3. Mechanism and Rationale

The therapeutic rationale for OR rests on the radiosensitivity of orbital lymphocytes and fibroblasts and on the anti-inflammatory effects of low-dose ionizing radiation. Low doses functionally modulate inflammatory cells, particularly lymphocytes, and have been proposed to act through anti-inflammatory and immunomodulatory pathways [4]. At a mechanistic level, single fractions at or below 1 Gy are anti-inflammatory through effects on leukocyte–endothelial adhesion, NF-κB signaling, and inducible nitric oxide synthase, a profile distinct from the pro-inflammatory effects of high-dose radiation [5].

The cellular substrate explains why timing matters. Active orbital tissue contains a CD4+/CD8+ T-cell and macrophage infiltrate driving a Th1 cytokine milieu (IFN-γ, TNF-α, IL-1β, IL-6), whereas inactive, fibrotic tissue lacks this infiltrate [6]. Radiation depletes the radiosensitive infiltrate and curtails fibroblast proliferation and glycosaminoglycan secretion, but it cannot reverse established fibrosis or fibro-adipose remodeling—orbital preadipocytes retain their adipogenic differentiation capacity even at doses that abolish their proliferation [5]. OR is therefore mechanistically suited to the early active phase and is expected to have little effect once disease has become fibrotic and inactive. This phase-dependence is central to interpreting both the trial evidence and the patient-selection criteria discussed below.

The same inflammatory substrate is also the target of IGF-1R blockade. In preclinical models (using the small-molecule IGF-1R inhibitor linsitinib rather than teprotumumab itself), IGF-1R inhibition reduces orbital T-cell and macrophage infiltration and suppresses IGF-1-driven fibroblast proliferation and hyaluronan secretion [7,8]. This suggests that OR and IGF-1R blockade act partly on overlapping inflammatory pathways rather than as wholly distinct modalities, although the inference is indirect and its relevance to human disease is unproven.

## 4. Radiotherapy Technique and Dose

The conventional regimen delivers a cumulative 20 Gy per orbit in 10 fractions of 2 Gy over two weeks [4,9]. Dose reduction and alternative fractionation have been explored: a lower total dose of 10 Gy has been reported to be of comparable effectiveness, and a protracted ultra-low-dose schedule of 1 Gy weekly over 20 weeks (20 Gy total) was at least as effective and better tolerated than the conventional two-week course in a randomized trial (a therapeutic response in 67% versus 55%, with radiation conjunctivitis in 0% versus 36% of patients), possibly reflecting a greater immunomodulatory effect at the lower fractional dose [4,9,10]. Technique has also evolved. Compared with lateral opposing fields and three-dimensional conformal radiotherapy, intensity-modulated radiotherapy (IMRT) achieves superior target conformity and homogeneity (*p* ≤ 0.007 for each comparison in a planning study) and better covers the anterior extraocular-muscle insertions while sparing the lens, retina, optic nerve, and lacrimal gland [11]. These dosimetric gains translate into a favorable long-term safety profile in clinical series [12]. Volumetric modulated arc therapy offers further organ-at-risk sparing; non-coplanar variants (HyperArc) improve the conformity index and steepen the dose gradient relative to standard arcs, while fixed-beam IMRT retains a slight homogeneity advantage [13,14].

**Figure 1:**
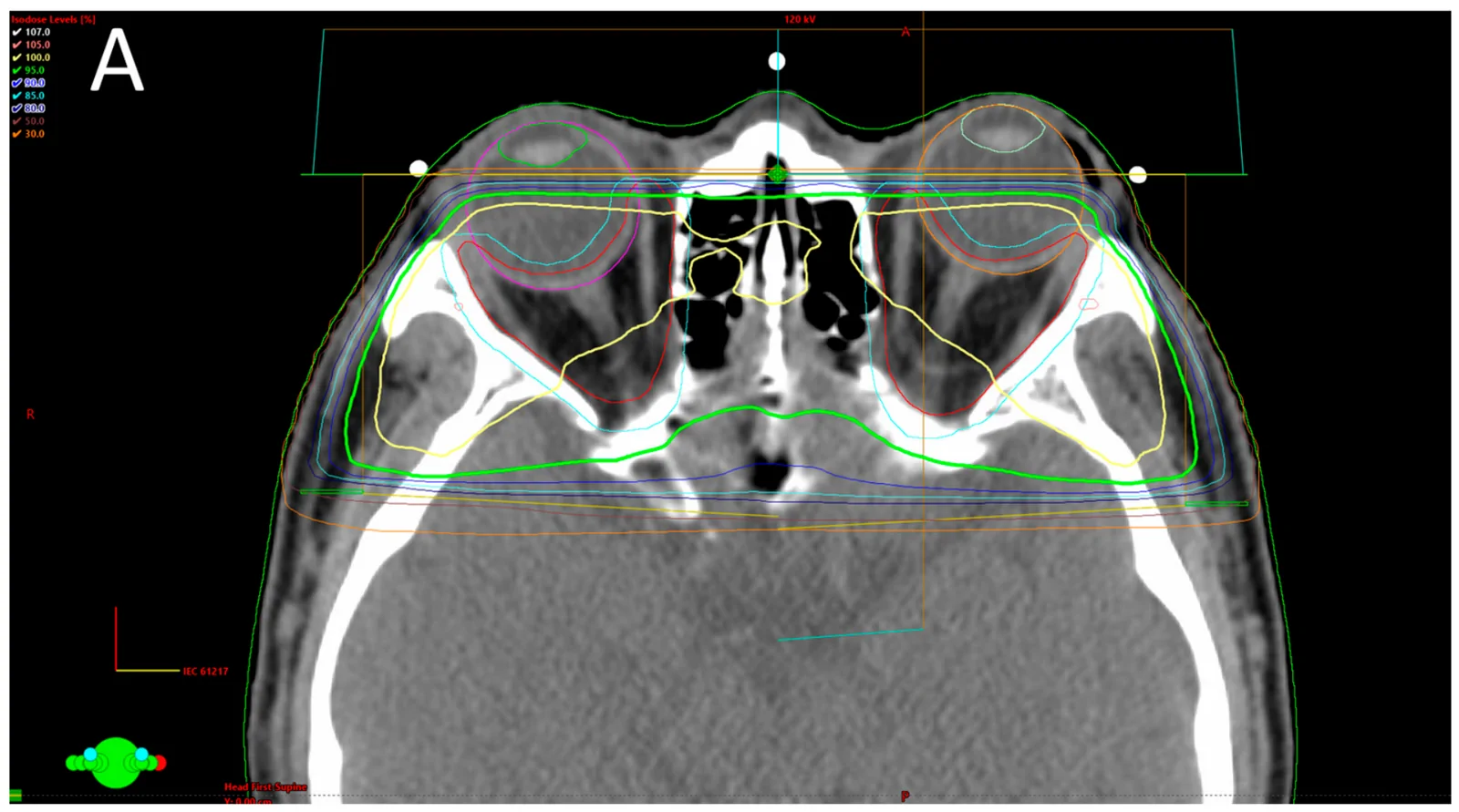

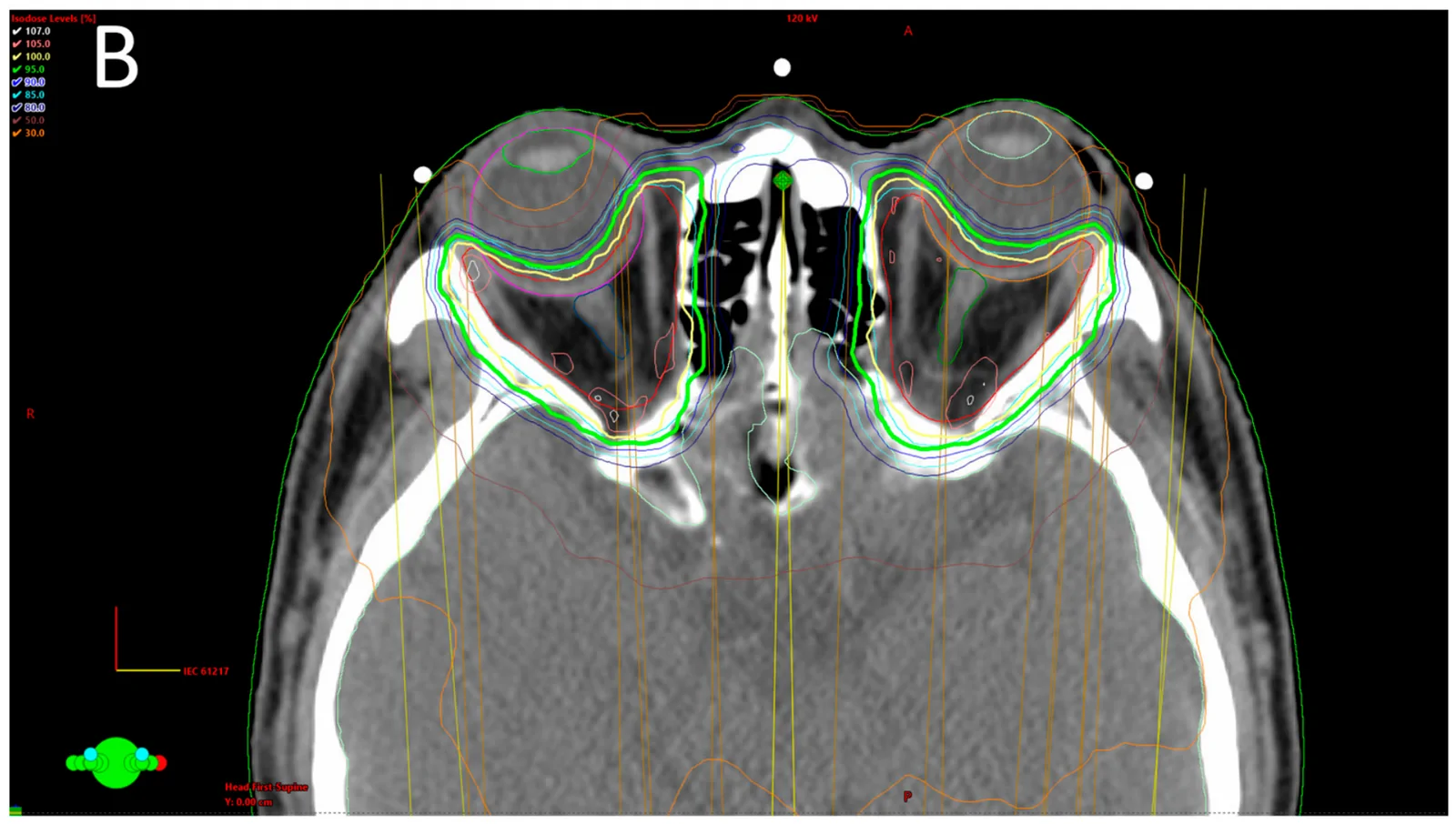
Example orbital radiotherapy plans prepared on the same head computed-tomography dataset, both prescribed 20 Gy in 10 fractions. (**A**) Three-dimensional conformal radiotherapy using a bilateral opposing pair of lateral fields. (**B**) Volumetric modulated arc therapy. Isodose lines are displayed as percentages of the prescribed dose (107, 105, 100, 95, 90, 85, 80, 50, and 30%), and the 95% isodose is shown as the thick green line. Both plans were generated with the Eclipse treatment planning system v18.0 (Varian Medical Systems, Palo Alto, CA, USA).

Figure 1 shows example plans for the two approaches, prepared on the same computed-tomography dataset at a prescription of 20 Gy in 10 fractions: a bilateral opposing pair of lateral fields (Figure 1A) and a volumetric modulated arc therapy plan (Figure 1B). The opposed pair delivers a single slab of dose that crosses the midline, whereas the arc plan conforms to each orbit separately. No comparative clinical study has shown that this dosimetric advantage alters efficacy, so the choice between the two rests on organ-at-risk sparing and departmental resources rather than on expected response. Modern planning reduces lens dose but does not eliminate it. In the same three-technique planning study, at a prescription of 10 Gy in 10 fractions, the mean lens dose was 5.7 Gy with three-dimensional conformal radiotherapy and 4.1 Gy with IMRT, against 3.1 Gy with lateral opposing fields, whose anterior block spares the lens at the cost of covering the muscle insertions [11]; a clinical IMRT series prescribing 20 Gy held the lens dose below 7 Gy, with cataract in 2 of 116 patients (1.7%) [12]. Conformity and lens sparing are therefore distinct objectives, and cataract remains a recognized late effect that improved technique has not abolished. Dose cannot be reduced without limit, however: an extreme low-dose schedule (4.8 Gy total) produced similar early palliation to 20 Gy but a markedly higher retreatment rate (13.1% versus 1.7%) [15], a caution relevant to the shortened regimens now under study. Two gaps in the evidence deserve explicit mention. Proton therapy remains theoretical for this indication, since no dedicated clinical outcome series in TAO has been reported, and data on orbital reirradiation are sparse.

## 5. Efficacy: The Randomized-Trial–Guideline Discordance

The pivotal randomized evidence is sobering. In the double-masked trial reported by Mourits and colleagues, patients with moderately severe Graves’ orbitopathy received either 20 Gy in 10 fractions or sham irradiation; a successful qualitative outcome at 24 weeks occurred in 18 of 30 (60%) irradiated versus 9 of 29 (31%) sham-treated patients (relative risk 1.9, 95% CI 1.0–3.6, *p* = 0.04) [16]. Tellingly, this advantage was driven by improvement in diplopia and ocular elevation, not by reduction in proptosis or eyelid swelling [16]. The result should be read with caution: the trial was small (*n* = 60) and single-center, and the confidence interval for the relative risk reached down to approximately 1.0, leaving the overall effect of borderline statistical significance. Across the body of sham-controlled randomized trials, OR has not been shown to be superior to sham for proptosis, lid fissure, or soft-tissue change [16,17]; these trials were themselves heterogeneous in severity, disease duration, and prior steroid exposure, which limits firm conclusions in either direction. The strongest single argument against OR comes from the Mayo Clinic trial, an internally controlled study (one orbit irradiated, the other sham, with crossover at six months) that found no clinically or statistically significant difference on any primary outcome at six months [18].

These individual trials have been formally synthesized. The 2012 Cochrane review pooled five randomized trials (244 participants) and found OR more effective than sham on a composite success outcome (risk ratio 1.92, 95% CI 1.27–2.91), with the benefit again concentrated in diplopia and motility; it found no difference between OR and glucocorticoid monotherapy, and noted that the included trials provided no long-term safety data and did not report radiation retinopathy [19]. A larger meta-analysis (33 trials, 1367 patients) likewise found no advantage of OR over sham on the clinical activity score, yet a clear superiority for diplopia (odds ratio 4.88, 95% CI 1.93–12.34) and for the combination of OR with corticosteroids over either modality alone (standardized mean difference −1.05, 95% CI −1.62 to −0.48) [20]. Two caveats temper these pooled estimates: the favorable diplopia and composite signals rest on only two trials each, so the narrow confidence intervals overstate precision, and the controlled-trial evidence base predates teprotumumab and modern outcome measures.

Evidence accumulated since the randomized era is observational but consistent in direction. In a retrospective series of 62 patients with steroid-resistant disease treated with 20 Gy in 10 fractions, the clinical activity score fell from 3.3 at baseline to 0.2 at 12 months, with disease reactivation in only two patients [21]. A separate cohort reported improvement at six months in 68.8% of patients with late-active disease and 80.0% of those with early-active disease, indicating that benefit is not confined to the earliest weeks of the active phase [22]. Contemporary safety data point the same way: a nationwide claims-based cohort of 1108 irradiated patients found radiation retinopathy in 5.7% and new cataract in 6.0% [23], and a linear-accelerator series reported no radiation retinopathy among 97 followed patients, including 19 with diabetes [24]. None of these are controlled, and none can establish efficacy against a sham comparator; their value is in showing that the direction of effect and the safety profile seen in the trial era have persisted with modern technique and in contemporary practice.

How, then, do guidelines continue to recommend OR? The discordance is best resolved by attending to what OR does and does not do. The randomized endpoints that “failed” (proptosis and soft-tissue swelling) are not the endpoints OR is mechanistically expected to improve, whereas the endpoint that improved, motility/diplopia, is precisely the indication that guidelines specify [1]. Differences in patient selection (disease activity and timing) and outcome definition further explain the apparent contradiction. Read this way, the trial data and the guidelines are largely reconcilable, though the reconciliation is necessarily interpretive, since the randomized trials were designed with proptosis as a primary endpoint and did not meet it. OR is best understood as a motility-directed, activity-dependent intervention rather than a proptosis-reducing one. The strongest skeptical position deserves a direct answer. Some authors have argued that the evidence for OR is too weak to justify its routine use outside compressive optic neuropathy [25]. That view is a useful corrective against overstatement, but it understates the consistent signal (across two of three sham-controlled trials and multiple comparative series) for a motility benefit, which is precisely the indication the guidelines retain.

## 6. Combination with Glucocorticoids

OR is rarely used as monotherapy, but the strength of the combination evidence depends on which glucocorticoid route is considered. For oral glucocorticoids, randomized trials have shown that the combination of OR and oral steroids is more effective than either modality alone, supporting a genuine additive effect [26]. For intravenous glucocorticoids, however, the picture is weaker: no randomized trial has demonstrated that adding OR to intravenous steroids is superior to intravenous steroids alone, and the supportive data are retrospective—for example, a severity-weighted response of 61.1% with combined intravenous steroids plus OR versus 46.1% with intravenous steroids alone [27]. The combination retains a particular role in patients with an incomplete or absent response to a first course of steroids; a recent series in steroid-resistant disease reported a large reduction in clinical activity score (from 3.3 to 0.2 at 12 months) with reactivation in only two of 62 patients [21]. This pattern explains why guidelines frame OR as an adjunct to glucocorticoid therapy rather than a replacement for it. The incremental value of OR over intravenous steroids alone remains an open question.

## 7. Patient Selection, Predictors of Response, and Dysthyroid Optic Neuropathy

If OR is an activity-dependent treatment, then selecting genuinely active disease is decisive. Clinical activity score captures inflammation only imperfectly, and quantitative orbital magnetic resonance imaging can supplement it. Because T2 relaxation time tracks free-water content, T2-mapping and T2-weighted STIR sequences index active inflammatory edema in the extraocular muscles: a pre-treatment STIR signal-intensity ratio above ~3.6 predicted steroid resistance (odds ratio 1.862, *p* = 0.040), though on its own it discriminated only modestly (area under the curve ~0.67), with the clinical activity score performing somewhat better (~0.79); in a separate cohort, a high minimum T2 relaxation time combined with short disease duration predicted steroid responsiveness (area under the curve ~0.82) [28,29]. When used this way, imaging allows the clinician to direct a patient toward early combination therapy or radiotherapy rather than wait for empirical steroid failure. Among treated patients, baseline optic neuropathy and a high thyroid-stimulating antibody titer have each predicted a worse relapse-free outcome after OR plus steroid pulse [30].

Disease duration is less limiting than once assumed. In a series of moderate-to-severe active disease, OR produced improvement at six months in 68.8% of patients with late-active disease versus 80.0% with early-active disease, lower in the late group but far from negligible, supporting selection by inflammatory activity rather than by an arbitrary duration cutoff [22].

Dysthyroid optic neuropathy (DON) is the indication in which even skeptics concede a possible role for OR, and where the modalities now collide. Retrospective data suggest that prophylactic OR in active disease reduces progression to compressive optic neuropathy [31]. Teprotumumab has entered the same space: in multicenter and pooled real-world series of DON, most patients reported improved visual acuity (objective improvement in roughly 70% in one early study), though the data are small and uncontrolled [32,33]. Surgical decompression remains the definitive intervention when medical therapy fails. No trial has compared OR, decompression, and teprotumumab head-to-head for DON, so the hierarchy among them remains a matter of judgment rather than evidence.

## 8. Safety and Contraindications

The principal late risks of OR are retinal and lenticular. The risk of definite radiation retinopathy is reported as 1% to 2% within 10 years of treatment [17], although a large nationwide claims-based cohort recently reported a higher figure of 5.7%—a discrepancy the authors attribute partly to case-finding by diagnostic code rather than by symptomatic presentation [23]; “possible” retinopathy (one or more microaneurysms or hemorrhages) has been reported in up to about 21% [17]. Cataract was newly diagnosed in approximately 6.0% of patients in that nationwide cohort [23], with older long-term series reporting rates of roughly 10% [34]. Reassuringly, long-term follow-up has not demonstrated an increased risk of secondary malignancy or premature death attributable to OR [17,34]: no orbital-field tumors were detected on long-term imaging and survival matched population norms. A cohort of 575 patients with at least three years of follow-up put the radiation-induced meningioma risk at 0.17% and the hematologic-malignancy risk at 0.7%, within the theoretical 0.3–1.2% range [35]. Still, the available person-years remain limited and the latency of radiation-induced malignancy is long, so a small risk cannot be excluded.

These data shape patient selection, with some nuance. EUGOGO regards pre-existing diabetic retinopathy or severe hypertension as an absolute contraindication to OR, and diabetes without retinopathy as a relative one, because of additive microvascular injury; given the latency of radiation-induced malignancy, OR has also traditionally been avoided in patients younger than 35 years [26,34]. More recent large-cohort data temper both points: diabetes alone was not a significant risk factor for radiation retinopathy (elevated fasting glucose and a short interval from diagnosis to radiation were), and patients under 35 showed no excess of retinopathy—leading current authors to advise individualized, counseled decisions rather than a fixed age cutoff [23]. A contemporary linear-accelerator cohort reinforces this trend: no radiation retinopathy occurred among 97 followed patients, including 19 with diabetes, although pre-existing diabetic retinopathy and uncontrolled hypertension remained exclusion criteria [24]. Careful candidate selection (active disease, motility involvement, and attention to retinal vascular and glycemic status, including age, pre-existing diabetic retinopathy, and severe hypertension) both maximizes benefit and minimizes harm.

## 9. Orbital Radiotherapy in the Era of Targeted Biologics: Repositioning

Teprotumumab produces marked improvements in proptosis and diplopia (a proptosis response of 83% versus 10% with placebo in the OPTIC trial [3]): effects that OR does not reliably achieve for proptosis [36]. Taken alone, this might argue for replacement. Yet, real-world experience tempers the comparison, and its durability is debated. One retrospective analysis of 21 patients reported the proptosis response falling from 84% to 57% by one year, with a sustained response in only about one-third of patients and late inflammatory flares, alongside substantial cost and otologic adverse effects such as tinnitus and hearing loss [37]; other real-world series report considerably lower recurrence rates [38]. Whatever the precise rate, off-treatment relapse is a recognized limitation.

A fair comparison must, however, hold OR to the same standard, and on direct comparative-effectiveness grounds, OR fares modestly. In a Bayesian network meta-analysis of randomized trials in active moderate-to-severe disease, OR ranked seventh of eight effective options, below teprotumumab, mycophenolate (with or without intravenous steroids), rituximab, azathioprine, and intravenous glucocorticoids, and above only oral glucocorticoids; for diplopia specifically, however, no significant difference was detected among OR, teprotumumab, rituximab, and intravenous glucocorticoids [39]. That diplopia comparison is an indirect, low-power estimate (an absence of demonstrated difference rather than proven equivalence), but it is consistent with OR’s particular strength lying in motility rather than in proptosis. OR is also not free of its own limitations: its onset of benefit is slower than that of steroids, and a substantial proportion of patients still require rehabilitative strabismus or decompression surgery despite treatment. In the Mourits trial, only 25% of irradiated patients were spared additional strabismus surgery [16]. Where OR differs sharply from teprotumumab is in cost and accessibility. In a comparative analysis of treatment cost and quality-of-life impact, the mean treatment-related cost was USD 4316 for a course of OR against USD 386,424 for teprotumumab; teprotumumab produced the largest absolute quality-of-life gain (ΔGO-QoL 19.4 versus 6.6 for OR) but at USD 19,888 per unit of quality-of-life change, about 30-fold that of OR (USD 651) [40]. OR’s economic advantage, rather than the magnitude of its quality-of-life effect, is thus its strongest health-economic argument. Two qualifications should frame that argument. First, the evidence tiers differ and should not be conflated: the cost-per-quality-of-life comparison is a conference abstract that has not completed journal peer review, while the Japanese assessment is a health-technology-assessment report that passed an agency process but not journal peer review. Neither is a peer-reviewed cost-effectiveness study in the conventional sense, and no such study yet compares OR with teprotumumab directly. Second, economic findings do not transfer across health systems unaltered, because unit costs, reimbursement rules, and the availability of each modality differ. Readers may find it useful to separate three questions that the available figures answer to different degrees: cost per quality-adjusted life-year, which speaks to value for money for an individual patient and is the only one addressed by the data above; budget impact, which speaks to affordability at system scale and can diverge sharply, since a treatment may be cost-effective per patient yet unaffordable across a population; and equity of access, which asks whether the less costly modality is in practice the only reachable one for a given population. Robust, multi-country cost-effectiveness analyses comparing OR, teprotumumab, and other biologics, using harmonized outcome measures and explicit budget-impact modeling, are therefore a key research priority. A formal Japanese health-economic assessment reached a similar conclusion, reporting an incremental cost-effectiveness ratio for teprotumumab—versus combined intravenous glucocorticoids and OR—of approximately JPY 9.4 million per quality-adjusted life-year (ICER JPY 9,355,796; incremental cost JPY 17.5 million for 1.87 additional QALYs) [41].

The safety ledger reinforces the point. Real-world, propensity-matched data link teprotumumab to a higher risk of new audiologic adverse events—a composite of tinnitus, sensorineural hearing loss, hypoacusis, hyperacusis, autophony, and Eustachian-tube dysfunction—with a relative risk of 2.85 (95% CI 1.94–4.20); irreversible sensorineural hearing loss has been reported, and OR carries no comparable risk [42]. Other biologics broaden the menu rather than settle it: the IL-6 receptor antagonist tocilizumab outperformed intravenous methylprednisolone for disease inactivation in a small randomized trial reported so far only in abstract form [43]. The tocilizumab trial and the Japanese cost model both await full peer-reviewed publication and are cited here as preliminary.

Framing the question as OR versus teprotumumab alone risks a false binary, because the biologic menu is widening and the agents act at different nodes. Teprotumumab blocks IGF-1R signaling on orbital fibroblasts and produces the largest proptosis effect of any available option. Tocilizumab blocks IL-6 receptor signaling and, in the randomized trial available so far only in abstract form, inactivated disease in 75% of patients at 12 weeks against 36% with intravenous methylprednisolone [43]. Rituximab depletes CD20-positive B cells and ranked fourth of eight active options in the Bayesian network meta-analysis, above both intravenous glucocorticoids and OR [39]. OR is unlike all three in that it targets no single molecule: it depletes the radiosensitive inflammatory infiltrate as a whole, which is why its effect is tied to disease activity rather than to a receptor’s expression, and why it is delivered as one finite course rather than as a continuing exposure. Read together, these profiles argue against ranking the modalities on a single axis. A widening menu makes phenotype- and resource-directed selection more necessary, not less: the relevant question for an individual patient is which mechanism matches the dominant clinical problem and which option is actually reachable, not which agent occupies the highest rank in a pooled analysis.

The policy context is shifting. Teprotumumab received European Union marketing authorization in June 2025, the first targeted TED therapy approved in the EU, which will intensify the access-and-cost debate that frames OR’s role [44]. Practice patterns also vary by region: in a society-endorsed Korean survey, 90.6% of respondents reported frequent use of OR and most reserved teprotumumab for glucocorticoid-refractory disease, constrained by cost and experience [45]—a reminder that, outside well-resourced settings, OR is not a historical relic but a workhorse.

**Figure 2:**
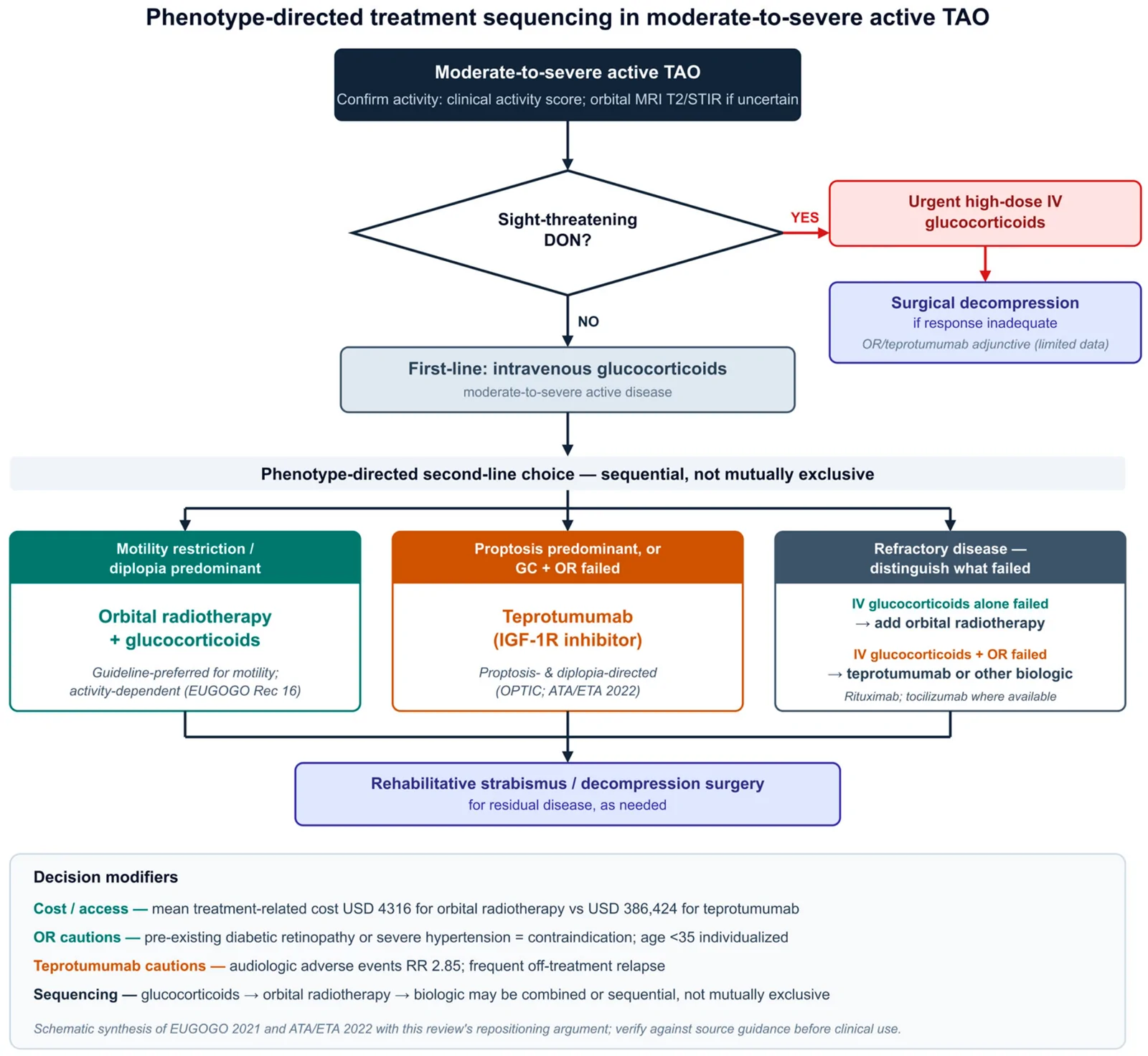
Proposed phenotype-directed sequencing of treatment for moderate-to-severe active thyroid-associated orbitopathy (TAO), synthesizing the 2021 EUGOGO and 2022 ATA/ETA recommendations with the repositioning argument advanced in this review. After confirming disease activity and excluding sight-threatening dysthyroid optic neuropathy (which requires urgent intravenous glucocorticoids and surgical decompression where response is inadequate), the dominant clinical phenotype guides second-line choice: OR combined with glucocorticoids is preferred where restricted motility or diplopia predominates, whereas teprotumumab is reserved for proptosis-predominant disease or failure of glucocorticoid-plus-radiotherapy. The modalities are sequential, rather than mutually exclusive. Listed cost, contraindication, and adverse-effect modifiers (including orbital–radiotherapy contraindications and teprotumumab ototoxicity) inform the choice. ATA, American Thyroid Association; DON, dysthyroid optic neuropathy; ETA, European Thyroid Association; EUGOGO, European Group on Graves’ Orbitopathy; GC, glucocorticoid; IGF-1R, insulin-like growth factor 1 receptor; IV, intravenous; MRI, magnetic resonance imaging; OPTIC, Treatment of Graves’ Orbitopathy to Reduce Proptosis with Teprotumumab Infusions in a Randomized, Placebo-Controlled, Clinical Study; OR, orbital radiotherapy; RR, risk ratio; STIR, short-tau inversion recovery; T2, transverse relaxation time; TAO, thyroid-associated orbitopathy; USD, United States dollar.

Against this backdrop, OR is better repositioned than retired. Its comparative strengths are a single-course, motility-directed effect, established long-term safety, wide accessibility, and far lower cost; its weaknesses are a limited effect on proptosis and a frequent residual need for surgery. A pragmatic algorithm therefore retains OR, combined with glucocorticoids, for active disease with prominent motility impairment, reserves teprotumumab for patients in whom proptosis reduction is the dominant goal or in whom glucocorticoid-plus-OR has failed, and considers the two as potentially sequential rather than mutually exclusive (Figure 2). The optimal sequencing of glucocorticoids, OR, and biologics is not yet defined by direct evidence.

## 10. Quality of Life

Because TAO is disfiguring and functionally disabling more often than it is dangerous, patient-reported quality of life is a central endpoint. The GO-QoL instrument separates visual functioning from physical appearance, and OR’s effect is phenotype-specific: a systematic review found that OR consistently improves the visual-functioning subscale, chiefly by stabilizing motility and diplopia, but does not meaningfully change the appearance subscale, since it cannot reverse established exophthalmos [46]. IGF-1R and IL-6 receptor biologics, by contrast, improve both subscales. This asymmetry is not a weakness of OR but a statement of what the modality is for. OR is an inflammation- and motility-directed treatment: its quality-of-life value is realized through function, and its case rests on delivering that function at a fraction of the cost.

## 11. Limitations and Future Directions

Three limitations of this review should be acknowledged. First, much of the contemporary evidence favoring OR (on cost, modern safety, and regional practice) is recent and, in places, drawn from conference abstracts or health-technology-assessment reports that are not yet fully peer-reviewed; these claims are presented as preliminary and should be confirmed as definitive sources appear. Second, because the synthesis was framed around the question of OR’s continued relevance, the newly assembled evidence weighs toward OR; the genuine and, for proptosis and appearance, superior effects of teprotumumab should not be understated, and the choice between modalities remains individualized. A third limitation follows from the literature rather than from our synthesis: disease activity was not defined uniformly across the included studies, with most using the clinical activity score with varying thresholds and a minority using orbital magnetic resonance imaging, so comparisons of response rates across studies carry an unquantified component of definitional heterogeneity.

The central uncertainties are now comparative and economic rather than mechanistic: there are no head-to-head trials of OR versus teprotumumab, no robust cost-effectiveness analyses positioning the two, and no validated biomarkers to select patients for one modality over the other. In the absence of such evidence, the honest position is one of clinical equipoise between modalities for many patients. Equipoise here carries its precise sense: genuine uncertainty within the expert community as to which option is superior for a given patient, rather than an absence of opinion or a settled hierarchy awaiting acceptance. It is not a counsel of inaction. In practice, five considerations narrow the choice for an individual: the dominant phenotype, since motility and diplopia point toward OR with glucocorticoids while proptosis and appearance point toward teprotumumab; disease activity, since OR acts on an inflammatory infiltrate that is present only in the active phase; contraindications and comorbidity, including pre-existing diabetic retinopathy and severe hypertension for OR, and hearing impairment, hyperglycemia, and pregnancy for teprotumumab; patient preference, weighing a single finite course against a multi-infusion regimen and weighing functional against cosmetic gain; and local resource constraints, which in many health systems determine whether the comparison arises at all. Where these considerations converge, the choice is usually clear despite the absence of head-to-head trials. Where they diverge, equipoise is real, and it should be named as such in the consultation rather than resolved by the clinician’s habit. Two specific gaps deserve emphasis. First, the long-term safety of OR rests on observational cohorts rather than trials; the Cochrane review explicitly noted that the randomized evidence base reported no long-term safety data and did not capture radiation retinopathy [19], so contemporary, conformal-technique safety data with extended follow-up are needed. Second, the dose question is being actively tested: the ongoing OraGO-1 randomized phase II trial compares a shortened schedule (10 Gy in 5 fractions) against standard 20 Gy in 10 fractions in patients with a documented poor response to intravenous methylprednisolone, with primary completion expected in April 2028 [47]. Prospective comparative studies, ideally head-to-head against teprotumumab and incorporating durability and cost endpoints, are the priority.

## 12. Conclusions

OR remains a legitimate, guideline-endorsed, motility-directed treatment for active moderate-to-severe TAO, most effective in combination with glucocorticoids and in appropriately selected patients, even as its overall efficacy continues to be contested. It is best understood not as a competitor to teprotumumab but as an accessible, low-cost modality whose particular strength lies in motility and diplopia rather than in proptosis, and whose limitations—a modest effect on appearance and a frequent residual need for rehabilitative surgery—should be stated as plainly as its advantages.

Three practical claims follow from this repositioning. OR should be retained, in combination with glucocorticoids, for active disease in which restricted motility or diplopia predominates, and wherever the cost or availability of biologic therapy constrains the alternatives. Teprotumumab should be reserved for patients in whom proptosis reduction is the dominant goal, or in whom glucocorticoids combined with OR have not succeeded. Clinical equipoise remains for the substantial group of patients with mixed phenotypes, for whom no head-to-head trial, no validated selection biomarker, and no robust comparative cost-effectiveness analysis yet exists; for these patients, the choice should be made explicitly based on phenotype, activity, comorbidity, preference, and access rather than by default.

Until comparative evidence exists, OR should be neither reflexively abandoned nor uncritically applied, but deployed for the indication that the evidence actually supports.

## Data Availability

No new data were created or analyzed in this study. Data sharing is not applicable.

## Abbreviations

The following abbreviations are used in this manuscript:

ATA	American Thyroid Association
CD4	cluster of differentiation 4
CD8	cluster of differentiation 8
CI	confidence interval
DON	dysthyroid optic neuropathy
ETA	European Thyroid Association
EU	European Union
EUGOGO	European Group on Graves’ Orbitopathy
GC	glucocorticoid
GO-QoL	Graves’ orbitopathy quality-of-life questionnaire
Gy	gray
ICER	incremental cost-effectiveness ratio
IFN-γ	interferon gamma
IGF-1	insulin-like growth factor 1
IGF-1R	insulin-like growth factor 1 receptor
IL-1β	interleukin 1 beta
IL-6	interleukin 6
IMRT	intensity-modulated radiotherapy
IV	intravenous
JPY	Japanese yen
MRI	magnetic resonance imaging
NF-κB	nuclear factor kappa B
OPTIC	Treatment of Graves’ Orbitopathy to Reduce Proptosis with Teprotumumab Infusions in a Randomized, Placebo-Controlled, Clinical Study
OR	orbital radiotherapy
PRISMA	Preferred Reporting Items for Systematic Reviews and Meta-Analyses
QALY	quality-adjusted life-year
RR	risk ratio
STIR	short-tau inversion recovery
T2	transverse relaxation time
TAO	thyroid-associated orbitopathy
TED	thyroid eye disease
Th1	T helper type 1
TNF-α	tumor necrosis factor alpha
USD	United States dollar
VMAT	volumetric modulated arc therapy
